# Professional education in PPPM as the realistic platform for the medicine of the future: EPMA takes action

**DOI:** 10.1186/1878-5085-5-S1-A12

**Published:** 2014-02-11

**Authors:** Olga Golubnitschaja

**Affiliations:** 1 Department of Radiology, Rheinische Friedrich-Wilhelms-University of Bonn, Germany

## 

Predictive, Preventive and Personalised Medicine (PPPM) is the new integrative concept of the paradigm change in healthcare that enables to predict individual predisposition before onset of the disease, to provide targeted preventive measures and create personalised treatment algorithms tailored to the person. The EPMA Journal (BMC, London, UK PubMed-indexed “open access”, http://www.epmajournal.com/ ) overviews the whole spectrum of PPPM relevant bio/medical scientific fields, publishing both review and research articles as well as position papers focused on

➣ patient needs

➣ scientific and technological excellence in PPPM

➣ professional consolidation

➣ implementation of innovative technologies in daily medical practice.

The expected impacts for the healthcare promotion are conducive to more effective population screening, prevention early in childhood, identification of persons at-risk, stratification of patients for the optimal therapy planning, prediction and reduction of adverse drug-drug or drug-disease interactions relying on emerging technologies, such as pharmacogenetics, pathology-specific molecular patters, sub/cellular imaging, disease modelling, individual patient profiles, etc. Integrative approach by PPPM is considered as the medicine of the future. Being at the forefront of the global efforts, EPMA promotes the integrative concept of PPPM among healthcare stakeholders, governmental institutions, educators, funding bodies, patient organisations and in the public domain (http://www.youtube.com/watch?v=SOwVS2Hh0KY). However, the main challenge of the concept performance is a creation of a robust educational platform for the stakeholders with the above listed spectrum of complementary activities as the multifunctional professional groups in the field.

**Figure 1:**
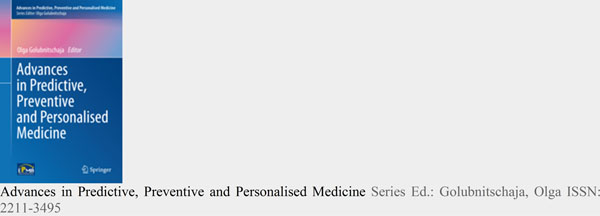
Professional education in Predictive, Preventive and Personalised Medicine gets effectively promoted by the book-series “Advances in PPPM”, Springer

Professional education gets effectively promoted by the EPMA through the book-series “Advances in PPPM”, Springer: http://www.springer.com/series/10051 (Figure 1).

In the years 2012-2013, following volumes have been released within the series:

- ***Healthcare Overview: New Perspectives***, Ed.: V. Costigliola, ISBN 978-94-007-4602-2

- ***New Strategies to Advance Pre/Diabetes Care: Integrative Approach by PPPM***, Ed.: M. Mozaffari, ISBN 978-94-007-5970-1

- ***Neurodegenerative Diseases: Integrative PPPM Approach as the Medicine of the Future***, Ed.: S. Mandel, ISBN 978-94-007-5865-0

- ***Drug Delivery Systems: Advanced Technologies Potentially Applicable in Personalised Treatments***, Ed.: J. Coelho, ISBN 978-94-007-6009-7

The books overview multidisciplinary aspects of advanced bio/medical approaches, scientific and technological innovation in corresponding fields. Integration of individual professional groups into the overall concept of PPPM is a particular advantage of this book series. Expert recommendations focus on the cost-effective management tailored to the person in health and disease. Innovative strategies are considered for standardisation of healthcare services. New guidelines are proposed for medical ethics, healthcare economy and marketing.

As its particular mission, EPMA Network considers supportive measures for the career promotion of young professionals in the PPPM related fields. A specialised section of young professionals EPMA-YPS has been created under the EPMA umbrella (http://www.youtube.com/watch?v=TwiUGaGashI). ***EPMA-YPS is keen to offer their expertise in helping young professionals*, *from a range of backgrounds*, *to identify useful international contacts*, *to provide support for them in applying for funding and to gain travel fellowships for research activities*** (EPMA-YPS Statutes, http://www.epmanet.eu/images/stories/pdfs/EPMA-YPS%20statutes.pdf ).

